# Studying and Learning Psychology During the COVID-19 Pandemic: A Mixed-Methods Approach on Students’ Perspectives of Psychological Well-being and Adjustment to Studying Online

**DOI:** 10.1177/14757257231169938

**Published:** 2023-04-27

**Authors:** Elida Cena, Paul Toner, Aideen McParland, Stephanie Burns, Katrin Dudgeon

**Affiliations:** School of Psychology, 1596 Queen's University, Belfast, Northern Ireland, UK

**Keywords:** Online learning, COVID-19, student well-being, blended learning

## Abstract

**Background:** The challenges presented by coronavirus disease 2019 (COVID-19) in higher education pressured learners and instructors to incorporate online emergent learning which presented several well-being and academic challenges to students. **Objective:** The purpose of this study is to examine the impact of studying online to students’ well-being. **Methods:** A mixed methods approach was followed for this study. Eighty students completed an online survey that measured their stress level of studying online, and 13 semistructured interviews were conducted at Queen's University Belfast. **Results:** Findings suggest that online learning under such circumstances increased students’ level of stress due to a number of perceived factors. Our findings also reveal the journey of student adjustment to online learning, reflecting the flexibility of blended learning as a long-term pedagogical strategy in universities, necessary for University's survival. **Conclusion:** As demonstrated in this study, after the initial difficulties of moving to online learning which had negative impacts on students learning and well-being, students subsequently adjusted to the online learning environment documenting students’ adaptability to a new learning environment and highlighting student resilience.

Even though the negative impacts of coronavirus disease 2019 (COVID-19) appear to be receding in Higher education as learning went back to its normal mode, the consequences of this are important to discuss for future potential pivots. In March 2020, after the World Health Organization (WHO) declared the COVID-19 outbreak to be a global pandemic, “stay at home” orders were issued in countries across the world, and the pivot to online learning began for academics and students. Whilst online learning has featured in Higher Education Institutions (HEI's) for many decades, facilitated in recent times by innovations in smartphone technology, high-speed data access and interactive online learning models, the evidence of its efficacy is largely divided. However, empirical studies have suggested that online learning can be more challenging for a number of reasons (Coman et al., 2020; Lemay et al., 2021; She et al., 2021; Zheng et al., 2021). At the onset of the Pandemic, HEI's were faced with an immediate need to deliver their degree programs online. In Northern Ireland, for the remainder of the 2019/2020 academic year and 2020/2021 academic year, lectures were prerecorded and asynchronously delivered via virtual learning environments and any face-to-face research activities were adapted to use the online research method. This mixed methods study will explore Psychology students’ learning experiences during the Pandemic and examine how the pandemic has impacted their general sense of well-being. In this study, qualitative and quantitative data will be used to understand students’ experiences of learning, socialization, and the perceived stress and challenges of studying during the pandemic. Furthermore, mixed methods will be used to understand the role of social support in the process of adjustment. With this in mind, the current paper will begin by reviewing the factors as they relate to challenges or positive opportunities associated with this transition and drastic change in education delivery.

## Challenges of Online Learning During the COVID-19 Pandemic

### Academic Support

The COVID-19 pandemic highlighted that HEIs need to enhance their capacity to deliver flexible long-lasting education. At an institutional level, this sector was faced with challenges around how they typically provide academic support to students. This was an all-encompassing issue that relies on a holistic approach from curriculum re-design and development to consideration of a very different educational environment that became increasingly remote for student learners (Naidu, 2021). Central to the challenges facing institutions was the support required to sustain meaningful student engagement (Wingo et al., 2017). Recent literature clearly highlights that institutional support was vital to the success of transitioning to online learning (Alqahtani & Rajkhan, 2020; Davies et al., 2020; Hartshorn & McMurry, 2020; Kara et al., 2020). The different support improvements ranged from providing clarity on plans and decisions as they evolved (Davies et al., 2020), providing support resources for faculty and students struggling with transitional arrangements to online learning (Hartshorn & McMurry, 2020), and paying for subscriptions for popular online platforms used institutionally (Kara et al., 2020).

While the education response that most institutions provided during this period on implementing remote learning strategies were intended to reach all students, not all students had equal access to educational resources and digital literacy. This raised concerns about educational inequalities regarding access to learning among students who had no access to learning facilities during this period (Coleman, 2021). Specifically, there are concerns about a digital divide, resulting in digital inequality, with already disadvantaged students being the most affected. Access to ICT was crucial for student learning and motivation during this period (Al-Kumaim et al., 2021; Jena, 2020). In places where learners had limited access to the internet, such as Pakistan and India, it was not possible for many students to effectively complete their university courses (Adnan & Anwar, 2020; Faturoti, 2022). In addition to access, students’ own digital competence is also seen to make a difference in learning. A study by Heidari et al. (2021) found that student digital competence was positively and significantly correlated with student engagement during the COVID-19 pandemic; students who felt confident with using e-technology tended to engage more with online learning. Together, these studies provide evidence that unequal access to the internet and learning technology coupled with varied student digital competence shows that the Pandemic has highlighted the intra-digital divide between countries and students, indicating the need the understand the lessons that can be drawn from this experience as we move forward.

### Social Interactions

In keeping with these challenges, and switching to a student-focused lens, it is important to consider the social factors that were exasperated by the move to online learning. Research has shown that the COVID-19 pandemic brought about a higher prevalence rate of social isolation and loneliness among young people when compared to older adults (Barreto et al., 2021). Various studies have estimated that between 38% and 50% of young people aged 18–24 years old experienced higher levels of loneliness during the lockdown in the UK (Bu et al., 2020), with women having higher odds of experiencing loneliness than men (Losada-Baltar et al., 2021; Salo et al., 2020). This trend has extended to student populations with qualitative studies providing a unique insight into students’ perceptions of social support during the COVID-19 pandemic. For instance, Mucci-Ferris et al. (2021) investigated the positive and negative experiences of undergraduate university students in the United States during the pandemic. Researchers found that many students shared negative aspects related to their social circles and experiences during this time. Not being able to see or interact with peers or attend classes was a prominent negative aspect for these students (Mucci-Ferris et al., 2021). Indeed, Alexiou-Ray and Bentley (2015) posited that the greatest challenge of online teaching is creating a sense of community and engagement that belies the distance created by technology. Despite the pivot to online learning presenting the opportunity to lean into this, we still lack a solid consensus on the best methods to build a learning community online as opposed to in a traditional classroom (Foster et al., 2021). It seems timely to develop an understanding of why this might be the case from students who now have first-hand experiences as a way to increase the level of preparedness for future potential pivots.

### Perceived Stress During the Pandemic

Another significant factor to consider is the psychological construct of perceived stress of individual students. Initial cross-cultural evidence of the psychological well-being of students during the pandemic showed an increase in stress and a decrease in the mental perceived stress of students in the UK (Savage et al., 2020). Even though perceived stress during the period of COVID-19 may be related to several factors, increases in student loneliness, anxiety, and stress were also reported in students studying in Switzerland (Elmer et al., 2020) and an increase in depressive symptoms in Italy (Meda et al., 2021). Another study that recruited 786 students attending an Australian university found that 34.7% of students reported a sufficient level of stress, while 33.8% showed low perceived stress and 31.5% very low perceived stress while studying during the pandemic (Dodd et al., 2021). The restrictions and the stress that were associated with the pandemic have put students at a greater risk of developing mental health issues. Some of the predicted factors influencing higher levels of distress among students were associated with the pandemic lockdown, fear of virus contraction, disruptive changes in health behavior and routines, lower levels of exercise, worsening of personal relationships, increase in tobacco use, unemployment, and financial concerns (Chen & Lucock, 2022; Farris et al., 2021; Schmits et al., 2021). More specifically, among the stressors that have influenced the anxiety levels of students in Bangladesh are frustrations generated by the lockdown conditions, economic situation, academic delays, and social support (Dhar et al., 2020). There is also an increase in aggressive behavior and internalization and externalization of problems during the lockdown (Parola et al., 2020).

Among the factors contributing to this poor picture of the psychological well-being of students during this period, were the levels of exhaustion inherent in online learning, with burn-out being associated with negative educational aspirations and outcomes (Güngör & Sari, 2022; Taylor & Frechette, 2022). Student perceptions of an increase in workload were particularly acute during this pivot to online learning. Yang et al. (2021) report that academic workload was positively correlated with perceived stress and negatively correlated with mental health during the COVID-19 pandemic among Chinese students. Researchers have linked these findings to difficulties students faced with managing their time independently and the inability to study in their home environments (Yang et al., 2021). Related to this, research has shown that a lack of motivation and self-regulation skills in online learning may result in students spending extra time completing assessments and having overall poor-quality work (Albelbisi & Yusop, 2019).

## Opportunities for Online Learning During the COVID-19 Pandemic

### Adjustment to Learning

Despite a challenging and negative picture presented thus far in the emerging pandemic literature around student experiences, the pandemic compelled faculty members and students to adapt their ways of teaching and learning. At an institutional level, educators were tasked with rethinking how curriculums and degree programs are delivered in HEI's using digital tools, modernizing pedagogical practices as we know them and widening accessibility for students. For instance, in a study that investigated students’ attitudes toward e-learning, Lochner et al. (2020) revealed that students’ attitudes towards this delivery mode were positive with enhanced learning experiences for students and engagement with lectures and lecturers. For example, students reported heightened levels of engagement with online learning by learning new skills related to remote working that will benefit them in their professional lives (Mucci-Ferris et al., 2021). Transferrable skills related to technology have also been found to be among the positive aspects of online learning (Mucci-Ferris et al., 2021). Emerging literature has highlighted that students have appreciated enhanced flexibility as one of the main benefits of online learning. These studies demonstrated that students benefitted from independent learning and the ability to study on their own time, convenience of studying from home (Almahasees et al., 2021; Fatoni et al., 2020). Many highlighted that the nature of online engagement was less intimidating than speaking in class, the low costs of not having to commute to campus and the time spent at home provided more time to sleep and gain energy which is important for their self-care and perceived stress (Zheng et al., 2021).

In addition, recent research has shown that university students’ resilience (Lee et al., 2021) and meaning in life (Arslan & Yıldırım, 2021) have potentially improved during the pandemic. When linked to online learning, findings from Lee et al. (2021) highlighted that this new generation of online learners seems to have a greater ability to cope with challenging situations and adjust to difficult situations than we would normally expect from adult students studying during prepandemic times. However, it is unclear how this improved resilience is translated into educational aspects, particularly adapting to an online learning context.

## The Present Study

Following these considerations, the empirical evidence suggests that the effects of the pandemic should be explored, and the findings can be used to understand the issues for future potential pivots (e.g., natural disasters, earthquakes, tsunamis, etc). While previous research has highlighted the positive and negative effects on student experience, this literature is either quantitative or qualitative, and to our knowledge, there is no literature that adopts a mixed method approach by combining the two approaches which can provide a more comprehensive view of student experiences during this period. As a research technique, the mixed methodology addresses questions that have eluded both quantitative and qualitative researchers applying their methods in isolation (Dickson et al. 2011). This research technique will give a better understanding of the challenges experienced by students during this period, as well as the adjustment process of students. Specifically, we examine the effects of online learning on academic support; students’ psychological perceived stress; student engagement; social interactions, and the process of adjustment to online learning. The current situation also appears to have changed the landscape of traditionally classroom-based learning; therefore, we examined the challenges and opportunities afforded by this online learning model. Two research questions were addressed:

1. What are the main challenges psychology students experienced with online learning during the pandemic?
  - How did such challenges relate to their perceived stress?
2. How did psychology students navigate and adjust to studying and learning during the Pandemic?
  - What was the role of student support during this period of adjustment?

## Methods

A mixed methods concurrent triangulation strategy was utilized in our study to strengthen the validity of findings through the congruence between the qualitative and quantitative results (Creswell, 2015), giving equal priority to each approach. The overall purpose of using the two methods was to examine the experiences of students studying during the pandemic, the factors that have affected their perceived stress, the process of adjustment, and the role of student support in this process. For this study, we chose the convergent parallel design in which qualitative and quantitative results are merged and compared with the aim of understanding the complexity of such experiences and the extent to which the quantitative and qualitative results converge.

Qualitative and quantitative data were collected separately within the same timeframe and participant cohort and the analysis started for both data sets after the data collection process had been completed (Fetters et al., 2013). As both methods have addressed the two research questions of this study, data were analyzed separately and then merged. In the final phase of the results, data were integrated together based on the categories that both sets of data identified and developed. This was done with the aim to highlight the concordance, consistency, and complementarity of the findings from the qualitative and quantitative elements (Dickson et al., 2011). Integration of analyses occurred at the interpretation stage whereby quantitative data were converted into “qualitized data” (that are textual descriptions of quantitative findings) to enable integration with the qualitative data (Stern et al., 2020). A semistructured interview schedule was used to explore the experiences of UK psychology students’ online learning and a quantitative survey. Data were collected from January to April 2021, and ethical approval was granted for each component of the overall study by Queen's University Belfast.

## Participants

*Qualitative:* Year 2 psychology students interviewed their psychology peers as part of their research methods training. Convenience sampling was utilized to recruit 13 Level 2 and Level 3 Psychology students. This method and sample were selected by researchers as the easiest way to access students during the COVID-19 period. Also, as data were collected by student researchers, selecting respondents who were “convenient” to them, enabled researchers to recruit peers they knew, which would help develop a trustful rapport with their participants and obtain richer data (Galloway, 2005).

*Quantitative:* A purposive sample of 80 students across all 3 years studying psychology at [name of university] were selected to take part, thus ensuring that year 1 students were included as the group of students who were entering their university life as remote learners. The table below provides demographic details of participants for the qualitative and quantitative study (Tables 1 and 2).

**Table 1.** Qualitative: Demographic Characteristics of Participants.

Pseudonym	Gender	Mature student (Yes/No)	Level of study
Anne	Female	Yes	Two
Daniel	Male	No	Two
Sarah	Female	No	Two
Mary	Female	No	Two
Polly	Female	No	Two
Penny	Female	Yes	Two
Jessica	Female	No	Two
Rose	Female	Yes	Two
Emily	Female	No	Two
Samy	Male	No	Three
Aoife	Female	No	Three
Hannah	Female	Yes	Three
Genny	Female	Yes	Three

**Table 2.** Quantitative: Demographic Characteristics of Participants.

Particapant	*n*	%
Gender		
Female	70	75
Male	18	22.5
Other	2	2.5
Age (in years)		
18–21	55	68.7
22–29	15	18.7
30–39	5	6.2
40–46	5	6.2
Year of Study		
Level 1	28	35
Level 2	35	43.7
Level 3	17	21.5

## Data Collection and Analysis

*Qualitative:* A semistructured interview guide was developed by students and checked by one of the authors to ensure that questions were designed to elicit in-depth responses closely aligned with the two research questions. They were open-ended and framed in a way that was easy to understand and that would allow participants to share their thoughts freely (Rubin & Rubin, 2012). This study was developed with the researchers’ role in mind and ensuring that the research process conformed to the standards of rigor and transparency in the data collection and analysis. In higher education research, academic staff must be aware of the power imbalances involved when involving students in research. In avoiding any such issues, all interviews were conducted by student researchers who knew participants and shared similar experiences with them. As insider researchers and empathy of the shared experiences, participants may have been more comfortable sharing detailed information or discussing issues with their peers who understand them better (Mercer, 2007).

**Figure 1.**
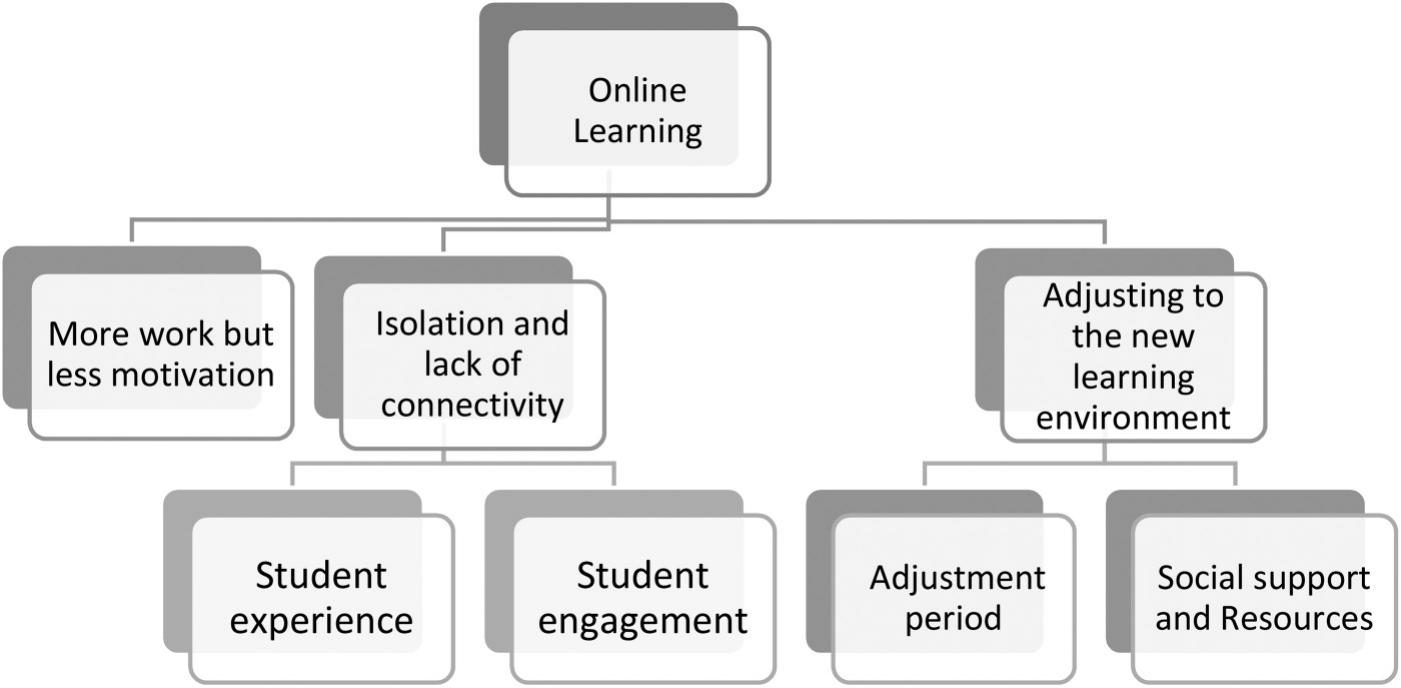
A representation of themes and subthemes.

Interview questions focused on uncovering the academic and technical challenges; questions on student experience and perceived social interactions; coping strategies and resources available to support online learning. Written and verbal consent were obtained from all participants prior to the study. Due to the pandemic, semistructured interviews were conducted individually on MS Teams and transcribed. Pseudonyms are used to de-identify participants mentioned in interviews. Data analysis followed the six-phase procedure outlined by Reflexive thematic analysis as a flexible theoretical method, suitable for mixed-methods analysis (Braun & Clarke, 2021). Using an inductive approach, two experienced qualitative researchers and authors of this paper, after familiarization, read each transcript independently and semantic and latent codes were identified. In this research, as academic staff, we had a good understanding of the issues surrounding the sector during this period but lacked the real insider perspective of how students experienced their learning, how their sense of belonging and well-being was impacted and the aspects of support that played a role during this period. Hence, to gain a better insight into students’ views, we ensured to sift through the data several times and represent the nuanced experiences of this student group. Codes were collated together into potential themes for each transcript, by combining similar codes which described some recurring patterns. We then discussed and progressed into the next step of identifying and developing themes. Figure 1 outlines the themes and sub-themes developed in the qualitative phase during the coding process. Theme construction was iterative and reflexive, with the two analysts discussing the findings regularly throughout the process to sense-check ideas or explore the multiple assumptions or interpretations of the data. The aim was therefore to achieve richer interpretations of meaning and enrich our understanding of the data rather than reaching a consensus (Braun & Clarke, 2021). In addition, in supporting credible and transparent analysis, we also held discussions and debriefings with the team of students who collected the data.

*Quantitative:* Students were invited to take part via social media, and an email invitation was sent to all undergraduate psychology students with Year 1 students receiving course credit for participating. The anonymous survey was hosted online through Qualtrics. Before gaining access to the survey, participants read an information page and provided their consent. Upon completion, participants were presented with a debrief page that detailed support services. The survey questions included the Perceived Stress Scale (PSS) (Cohen, 1988). PSS scores range from 0 to 40 with higher scores indicating higher levels of perceived stress.

Data were cleaned, checked, and analysed on IBM SPSS version 27. From 119 attempted completions, 35 answered no to the eligibility question about being a current Psychology student at [name of university]. Four participants answered yes to this question but then did not complete any further questions, therefore, only complete cases (*n* = 80) were included in the analysis. A total score was derived for the PSS by following the developers’ scoring instructions. Descriptive statistics and simple linear regression were conducted, with the independent variable being the quantity of workload (defined as whether workload has decreased a lot, stayed the same, or increased a lot on a 10-point scale) and the dependent variable the perceived stress score.

## Data Integration

After data were examined using the above analytical strategies, the data were integrated into the interpretation of the findings. This was done by comparing both qualitative and quantitative findings which allowed for an expanded understanding of findings from the analysis of both datasets when merged for interpretation (Moran-Ellis et al., 2006). Quantitative findings of identifying the factors that affected students’ learning and led to an increase in a perceived level of stress, and the role of social support were then reexamined across the themes which were identified from the qualitative analysis, as depicted in Figure 1 below.

## Results

To examine students’ barriers to studying during the Pandemic, the perceived stress, and the ways of adjusting to the new ways of learning and living, the qualitative findings identified three main themes^1^ as depicted in Figure 1. The first two themes are (a) More work but less motivation, (b) Isolation and perceived stress related to the first Research question and sub-question. These themes highlight the complexity of studying online during the pandemic, seeking to understand the main factors that have affected their psychological perceived stress. Factors fall into two sub-themes, academic-related and those focusing more on the student experience. The third theme is (c) Adjusting to the new learning environment which includes two subthemes (a) The adjustment period and (b) Social support and resources. Taken together, these aim to address the second Research question and the sub-question which focuses on identifying the ways students used to navigate through a challenging learning environment and the role of support from the academic and nonacademic staff in this process. The findings below will provide an in-depth of these themes starting initially with the qualitative findings and then the quantitative findings for each theme.

## More Work but Less Motivation

Findings suggested that the challenges of studying online are multifaceted, with the most common stressor being an increase in the workload in comparison to the previous year. Particularly during the initial phase of the transition to online learning, students reported being overwhelmed with the high volume of information and content and struggled to manage their time. Eleven of the participants reported being displeased with the fact that the university initially expected them to produce the same amount of work despite the changes and challenges posed by the new learning system and the ongoing public health threat students faced with COVID-19.

*One thing that I feel is quite unfair, is the level of work, and in terms of like support and, you know? I found that obviously times have changed and we're in very different situations, but we're still expected to produce the same amount of work even with things going on at home. (Anne)*

The abrupt transition to online required the quick incorporation of a new approach to learning and in general, students felt that online lectures took longer time to watch and go through. Nine participants perceived that the home environment was not a suitable place to study. Seven participants reported that the university should have provided clearer guidelines earlier and given longer extensions for the coursework. In addition, they reported feeling anxious about online exams, and highlighted increased expectations placed on them as a result of open book exams being implemented.

Another major challenge of studying online was reported to be a *lack of motivation*. Nine students found it difficult to motivate themselves, and often they procrastinated. As Sarah stated below:

*Most resources online and no access to the library have made it difficult to do the assignments. I am doing the bare minimum of what the course asks you to do.*

Online learning required a lot of self-discipline and careful planning which many students found challenging to engage with. Long hours in front of the computer every day, watching endless video recordings was described as a boring experience and led to a decrease in student motivation and unwillingness to learn.

These findings are supported by the quantitative findings which revealed that students perceived an increase in their workload since the move to online learning; on a scale of 1–10, with 1 indicating “decreased a lot,” 5 indicating “stayed the same,” and 10 indicating “increased a lot,” the mean score was 6.9 (*SD* = 2.55). The mean number of hours spent learning online per week was 20 (*SD* = 13.62). Survey results indicated that students faced overall well-being issues, particularly around stress and loneliness. When asked about the extent to which they felt the shift to online learning had affected their stress levels, on a scale of 1–10 with 1 indicating “not stressed” and 10 indicating “completely stressed,” the mean was 6.2 (*SD *= 2.57).

A simple regression analysis was also performed to assess whether the quantity of online workload predicted PSS scores. The regression model was statistically significant *F (*1,72) = 7.63, *p* = .007 with adjusted *R*^2^ indicating 8.3% of the variance in students’ perceived stress scores was accounted for by the quantity of workload. The regression coefficient (β = 0.946) indicated that an increase of one unit of workload corresponded to an increase of 0.946 in perceived stress score. In terms of frequencies, most students (66.7%) reported that the quality of their learning since the beginning of the pandemic had been negatively impacted; just under one-fifth (19.2%) felt it had stayed the same; and 14.1% felt it had been positively impacted.

### Isolation and Lack of Community

*Student Experience**.*** Findings suggest that social distancing appears to have increased students’ feelings of isolation, which was associated with loneliness, stress, and frustration of being on their own to do work.

*Yeah definitely, because the part I enjoyed most about Uni was going to classes and being there and engaging with others around me. That has just been completely removed, which makes it a very intense, really intense learning environment and you’re in the same space when you’re relaxing and working, so it's hard to separate the two, so it makes me more stressed. (Mary)*

As the above demonstrates, the absence of aspects that made University a special experience, such as interactions with others and being present in the University, appears to have led to students experiencing social isolation. In particular, stronger isolation was reported by first-year students, who did not develop any type of social interaction with other peers from the year group. Isolation and lack of social interaction were also reported by participants who had little social support, those who had issues with technology at home, and international students who also started to question the worth of their degree:

*All the time, it scares me especially being an international student, and then like you know sometimes it gets lonely and then …, you just be like why am I here? I just wanna go home. Do I need this degree kind of thing? (Samy)*

*Lack of Community**.*** For 11 students, there was a lack of community during the pivot to online learning which impacted student engagement. Although students learned how to adapt to MS Teams and navigate the online environment, student interaction with other peers “… that pulls you into that learning environment,” was missing. Students struggled to work on their own without the casual chats and discussions after the lecture. However, findings suggested that student engagement online was particularly challenging with students actively disengaging from group and thesis projects, “a lot of students I feel like they don’t feel accountable to contribute work.” There was also hesitation to ask questions online, as it was perceived as more formal and required more thought when passing a comment.

*I've avoided interacting online with anybody. I hate online meetings. Just the whole …, freezing and people talking over each other …, it's just … it's so awkward and awkward silences and I just felt uncomfortable, and I hated them. Hated the whole online experience (Aoife)*.

These findings were supported by the survey findings whereby almost all the students surveyed reported experiencing some degree of loneliness since the shift to online learning. Only 5.4% reported no loneliness since the shift; 41.9% reported “sometimes” feeling lonely; over half (52.7%) reported “often” or “always” feeling lonely. The extent of communication students had with other students reflects these reported levels of loneliness: Almost four-fifths (79.7%) reported having less communication with other students; 8.1% reported having the same level of communication as before; and 12.2% reported having more communication than before. A similar pattern, though not as extreme, was found for engagement with lecturers since the shift to online learning: half of the students (50%) reported decreased engagement with lecturers; 29.7% reported the same level of engagement as before, and 20.3% reported more engagement than before.

## Adjusting to the New Learning Environment

*Adjustment Period.* Participants’ accounts demonstrated they began to overcome “teething problems” shortly after the end of semester 1*,* which included technology-related difficulties and started a new phase of adjustment to the online learning environment. The most beneficial aspect of online learning was reported to be the level of autonomy students had to manage their own time as independent learners. Students who have shown a more positive attitude were those who had the infrastructure and quickly adopted by developing skills of being organized. The gradual familiarization with the online environment was shown by narratives of establishing a routine of dividing their own time when to study and when not:

*In the beginning, I probably found it harder, but now that I'm in a routine I think I kind of know, like when I should do this lecture and how long each one is going to be and having the same time for sure every week and stuff online. It hasn't really been that different to last year. Em, so I haven't found it that bad. (Jessica)*

Nine of the students indicated to have made changes to their lifestyle by setting up a routine that included regular exercise, listening to music, playing videogames, and notably they expressed a desire to be in outdoor spaces. From all this experience, it appears that students appreciate the flexibility of blended learning. Particularly for mature students, despite the challenges of having to juggle multiple roles, they reported enjoying the convenience, flexibility of studying, time and saving on traveling costs, “Not feeling like a student, in a positive way. It gives the freedom to study, work and take care of children” (Hannah)*.*

These findings were corroborated by our survey findings which revealed a range of ways in which students have had to adjust and adapt to online learning. At the time of the survey, the hybrid-model of teaching and learning (i.e., online and face-to-face teaching and learning) was the preferred learning format (66.7%), with a third (33.3%) preferring the online-only format—none reported face-to-face alone as their preferred format—a finding which underscores the huge adjustment that students had undergone in their learning experiences since before the pandemic.

*Support and Resources.* Students mostly demonstrated appreciation for the efforts and pastoral care of lecturers and personal tutors throughout the pandemic. They felt that lecturers provided responses in a timely manner and found ways to engage students actively in learning.

*Em, well they’ve definitely met all their targets on having everything uploaded on time and stuff. And I feel like they have gone the extra mile in making sure there are places for you to ask questions, if you need to. (Rose)*

Communication with lecturers during tutorials, which took place once on a weekly basis this year (previously fortnightly), encouraged consistent communication among students and increased student interactions. On many occasions, it was reported that lecturers provided additional discussions and sessions to support students with their learning. Students who had social network systems, those who lived at home, had support from their families and siblings reported being more positive about this experience. Maintaining relations with existing friends and other students from tutor groups via instant messaging or Zoom was a good source of support.

The survey findings also shed light on multiple aspects of the resources and support students needed to deal with the difficulties. Most students perceived some degree of support from the central university (only 10.8% reported receiving “no support,” and 18.9% reported receiving “maximum support,” with a mean of 3.2 (*SD *= 1.25) on a scale of 1–5 for support), and most students reported satisfaction (or better) with the level of helpfulness of their lecturers (6.8% of students reported lecturers being “not helpful at all,” while 23.0% reported lecturers being “extremely helpful,” with a mean score of 3.5 (*SD *= 1.19) on a scale of 1–5 for helpfulness.

## Discussion

This study examined the impact of COVID-19 on psychology students’ learning experience at [name of university]. We hope that the findings discussed in this study are useful for HE institutions in the actions they take in the process of preparedness toward future potential pivots. The study aimed to address two main research questions. The first was on the challenges students experienced with online learning during the pandemic, and the second question referred to the ways in which students were able to navigate through online learning and the role of other individuals and resources in this process. In contrast to previous research, methodologically, we centerd our study on understanding student experiences from a mixed-methods lens to get rich insight from the combination of the two methods which enabled an understanding of challenges and the dynamics of the adjustment processes for online learners.

*Academic and Social Concerns*. Both qualitative and quantitative data indicate that the initial experiences of moving to remote learning impacted negatively on their learning. The first theme on Increased work and lack of motivation revealed that students felt stressed and frustrated because of the consequences brought about by the pandemic. The quick transition to online learning exasperated this experience. These findings are in line with existing research conducted recently in other parts of the globe (Cohen et al., 2020; Yang et al., 2021). Moving online at short notice amplified student workload and time commitment, which could be due to the amount of reading required for online course delivery more broadly (Kaczynski & Kelly, 2004). Another challenge was related to the fact that students had to become autonomous learners, which necessitated developing quick time management skills. While research has previously established that academic workload is one of the main stressors for college students (Murff, 2005), the current situation with sickness or family stressors may have compounded the difficulty of adjustment to online learning.

Findings of the second theme revealed that students experienced isolation and loneliness which was identified as significantly related to their perceived stress. It was notable that one of the most important aspects of their student experience which was affected by online learning was social interaction. There was a lack of community in the online learning environment, which is strongly related to their perceived stress, which corroborates with earlier research (Masha'al et al., 2020; Yang et al., 2021). Our findings document that while studying online, students highlighted the difficulties of working individually, therefore recognizing the role of their peers as critical to their learning experience. Findings support previous research (Phirangee & Malec, 2017) regarding the need to foster connectedness and a strong sense of community in online learning, which underpins that establishing a social presence with peers and lecturers is necessary to decrease students’ feelings of isolation.

Adjustment and Support. The final theme provided valuable insight in showing that the move to online learning portrayed a journey for students. Over time most students adjusted to online learning and began to appreciate various aspects of online learning. Findings also revealed that not all students were impacted negatively or at least in the same way. Similar to previous research (Biwer et al., 2021), our students, coped and adjusted at a different pace and there were individual differences when adjusting to remote learning. It appears that there are different challenges for different students or student populations, depending on technology issues, academic integration, social integration, motivation, and support. In line with other research (Farell & Brunton, 2020) which highlights the need for students to develop their time management and organizational skills, students in our sample gradually learned how to manage their time by creating a systematic approach to study as well as balancing many other competing commitments of studying from home.

Our students appreciated many aspects of online learning by becoming better self-directed learners and progressively mastering content more efficiently and successfully. Findings highlighted the positive role of closer communication during tutorials, discussions, and online meetings which enhances peer interactions. Similar to other research on online learning, our students highlighted the benefits of having more freedom and access to learning content in a flexible manner and at their own pace of learning (Delaney & Fox, 2013). This included more freedom with time management and flexibility with their own pace of learning, for example, utilizing previous travel time as study time. For many students, it was reported to be cost-effective as it did not require learners to travel to be on campus. In this way, these findings suggest that students appreciated the blended learning mode with integration of elements of face-to-face and e-learning.

As the combination of face-to-face teaching with technology is greatly being recognized as an educational method that can increase the learning potential of students (Megahed & Ghoneim, 2022) the current findings support other research which suggests that HEIs should build institutional frameworks that enable the high quality of Blended learning models (Cobo-Rendon et al., 2022).

Several limitations need to be considered when interpreting the present findings. Although a mixture of undergraduate students participated in the study, only second and third-year students were recruited for the qualitative part of the study. Although recruited from the same cohort, due to the anonymous nature of the survey it was not possible to cross-reference quantitative and qualitative findings for individuals taking part. Future research should consider including not only psychology students but a more diverse sample of students from other degree programs, PGT, and international students. Those findings would allow for more bespoke assistance by HE, depending on the needs of the various student cohorts. This would be in line with previous research that highlights that different students have different needs and that institutions should look for various ways of supporting students (Daniel, 2020). Participants represent students at a university who may have followed a specific way of managing the situation of online learning during Covid, therefore, generalizations of the findings to other contexts need to be made with caution.

Studying online during the pandemic affected student life in several ways: academically, socially, psychologically and experiential factors that comprise the student life in higher education. What can be concluded is that after initial difficulties moving to online learning with negative impacts on students learning and perceived stress, students subsequently adjusted to the online learning environment highlighting student resilience and ability to adjust. As student mental health is greatly affected during the pandemic, support systems at the departmental level should encourage students to seek professional support provided by the university in facing potential distress. They should have access to additional training with practical exercises to help them gain skills in managing their time and assignment tasks. Identifying student needs and challenges experienced during the pandemic is a crucial step to ensure a smooth transition to the new, post-pandemic era in Higher Education that students are entering.

## Notes

1. Themes are discussed by using some quantifying language to show the prevalence across the data corpus. Nevertheless, by using numbers or quantifying language we do not in any way attempt to count instances as strength as per a content analysis.

## Appendix A

### Interview Guide

#### Educational Difficulties

1. Are there any challenges you have experienced at university related to the pandemic?

2. Talk me through what an average week of online learning consists of for you.

3. Do you have access to all the required resources?

4. What have been the main differences between online and traditional learning?

5. Has the change from traditional learning to online impacted your level of engagement?

#### Social Well-being

6. How have you felt about social interactions during this time?

7. Has online learning changed the way in which you interact with your peers? [In what way?]

8. Have your friendships with your group peers changed during this year compared to last year [how?]

9. How do you balance your time between study and personal time?

10. Tell me a little about your current living situation? [how has that helped or hindered online learning?

#### Psychological Well-being

11. What kinds of emotions have you experienced in relation to studying online? [do you experience loneliness, or independence?]

12. Would you say online learning has impacted your stress levels compared with traditional learning? [why?]

13. Do you have any activities or personal rituals that help you to cope with stress? [can you give me an example]

14. If you could speak to your younger self and prepare them for the move to online learning what advice would you give yourself to prepare for the change?

15. In what ways has the university supported you during this transition?

16. How do you think the university could best support you for the next semester?

Thank you very much for all the information you have provided, your participation has been valuable. Is there anything else you would like to add before we end?

### Appendix B: Quantitative Survey

Q1 Are you an undergraduate Psychology student studying at [name of University]?

Yes (1) No (2)

Q2 Please select what year of study you are currently in:

First (1) Second (2) Third (3)

Q3 What is your gender?

Male (1) Female (2) Other (3) Prefer not to say (4)

Q4 What is your age?

_________________

Q5 Prior to lock down in March 2020, have you had previous experience with online learning?

None (1) Yes - A Little (2) Yes - Some (3) Yes - A Lot (4)

Q6 Do you have any of the following responsibilities outside of education? (select all that apply)

Part-time Work (1) Family Duties (2) Carer Duties (3) Other (4) None (5)

Q7 How equipped are you for online learning?

Not Equipped (1) Moderately Equipped (2) Well Equipped (3)

Q8 Do you have access to WiFi at home or at your main place of online study?

No (1) No—I use mobile data (2) Yes—At home (3) Yes—At place of study (4)

Q9 Do you have access to a computer/laptop at home?

Yes (1) No (2)

Skip To: Q11 If Do you have access to a computer/laptop at home? = No

Q10 If yes, is this your own or shared computer/laptop?

My Own (1) Shared (2)

Q11 Do you have a suitable workspace? (E.g. access to a desk, comfortable chair, adequate space etc.)

Not Suitable (1) Reasonably Suitable (2) Very Suitable (3)

Q12 How many hours roughly do you spend studying online each week?

Insert the number of hours

_____________________

Q13 As a result of the shift to online learning, has your workload?

0 (0) 1 (1) 2 (2) 3 (3) 4 (4) 5 (5) 6 (6) 7 (7) 8 (8) 9 (9) 10 (10)

(0 = Decreased a lot, 5 = Stayed the same, 10 = Increased a lot)

Q14 Has the shift to online learning affected your ability to manage your workload?

Negatively Affected (1) Stayed the Same (2) Positively Affected (3)

Q15 Do you feel the quality of learning has been affected as a result of the shift to online learning?

Negatively Affected (1) Stayed the Same (2) Positively Affected (3)

Q16 Do you find the prerecorded lectures easier to follow than face-to-face lectures?

No (1) Much the Same (2) Yes (3)

Q17 In the last month, how often have you been upset because of something that happened unexpectedly?

0 = Never (1) 1 = Almost Never (2) 2 = Sometimes (3) 3 = Fairly Often (4) 4 = Very Often (5)

Q18 In the last month, how often have you felt that you were unable to control the important things in life?

0 = Never (1) 1 = Almost Never (2) 2 = Sometimes (3) 3 = Fairly Often (4) 4 = Very Often (5)

Q19 In the last month, how often have you felt nervous and “stressed”?

0 = Never (1) 1 = Almost Never (2) 2 = Sometimes (3) 3 = Fairly Often (4) 4 = Very Often (5)

Q20 In the last month, how often have you felt confident about your ability to handle your personal problems?

0 = Never (1) 1 = Almost Never (2) 2 = Sometimes (3) 3 = Fairly Often (4) 4 = Very Often (5)

Q21 In the last month, how often have you felt that things were going your way?

0 = Never (1) 1 = Almost Never (2) 2 = Sometimes (3) 3 = Fairly Often (4) 4 = Very Often (5)

Q22 In the last month, how often have you found that you could not cope with all the things you had to do?

0 = Never (1) 1 = Almost Never (2) 2 = Sometimes (3) 3 = Fairly Often (4) 4 = Very Often (5)

Q23 In the last month, how often have you been able to control irritations in your life?

0 = Never (1) 1 = Almost Never (2) 2 = Sometimes (3) 3 = Fairly Often (4) 4 = Very Often (5)

Q24 In the last month, how often have you felt you were on top of things?

0 = Never (1) 1 = Almost Never (2) 2 = Sometimes (3) 3 = Fairly Often (4) 4 = Very Often (5)

Q25 In the last month, how often have you been angered because of things that were outside of your control?

0 = Never (1) 1 = Almost Never (2) 2 = Sometimes (3) 3 = Fairly Often (4) 4 = Very Often (5)

Q26 In the last month, how often have you felt difficulties were piling up so high that you could not overcome them?

0 = Never (1) 1 = Almost Never (2) 2 = Sometimes (3) 3 = Fairly Often (4) 4 = Very Often (5)

Q27 Please select where you carry out the majority of your online study:

Own Home (1) Family Home (2) Student Accommodation (3) Library (4)

Q28 Are there many distractions in your place of study?

1 (1) 2 (2) 3 (3) 4 (4) 5 (5)

(1 = No distractions, 5 = Many distractions)

Q29 Do you feel the quality of student support has been affected as a result of the shift to online learning?

Negatively Affected (1) Stayed the Same (2) Positively Affected (3)

Q30 How helpful have your lecturers been in providing support while studying remotely?

1 (1) 2 (2) 3 (3) 4 (4) 5 (5)

(1= Not helpful at all, 5 = Extremely helpful)

Q31 Do you think there is sufficient support provided by the University for online learning?

1 (1) 2 (2) 3 (3) 4 (4) 5 (5)

(1 = Minimal support, 5 = Complete support)

Q32 Do you feel lecturer to student engagement has decreased, stayed the same, or increased due to the shift to online learning?

Decreased (1) Stayed the Same (2) Increased (3)

Q33 Has communication with other students been the same with online learning as it was with face-to-face learning?

Less Communication (1) The Same (2) More Communication (3)

Q34 Do you feel lonely or isolated as a result of online learning?

No (1) Sometimes (2) Often (3) Always (4)

Q35 What type of learning did you engage in before the outbreak of COVID-19? (hybrid = combination of both face to face and online)

Face to Face (1) Hybrid (2) Online (3)

Q36 Based on your previous learning experience, how worried did you feel about the shift to online learning?

1 (1) 2 (2) 3 (3) 4 (4) 5 (5)

(1 = Not worried, 5 = Extremely worried)

Q37 Based on your previous learning experience, did you feel capable to manage online learning?

1 (1) 2 (2) 3 (3) 4 (4) 5 (5)

(1 = Not capable, 5 = Completely capable)

Q38 How has the shift from face-to-face to online learning affected your stress levels while studying?

0 (0) 1 (1) 2 (2) 3 (3) 4 (4) 5 (5) 6 (6) 7 (7) 8 (8) 9 (9) 10 (10)

(0 = Not stressed at all, 5 = Moderately stressed, 10 = Completely stressed)

Q39 What is your preferred teaching method during the COVID-19 pandemic? (hybrid = combination of both face to face and online)

Hybrid Method (1) Online Method (2)

Q40 How do you find online learning?

1 (1) 2 (2) 3 (3) 4 (4) 5 (5)

(1 = Easy, 5 = Difficult)

Q41 How comfortable do you feel asking questions in online classes compared to face-to-face classes?

0 (0) 1 (1) 2 (2) 3 (3) 4 (4) 5 (5) 6 (6) 7 (7) 8 (8) 9 (9) 10 (10)

(0 = Less comfortable, 5 = No difference, 10 = More comfortable)
